# The Stomach

**Published:** 1869-11

**Authors:** 


### THE STOMACH

Is the source of a very large share of our animal enjoyment, if treated properly; but if allowed to fall into disease, life is rendered miserable, in spite of all the advantages that wealth or station can bestow. Eating largely and late, is the most common cause of the long catalogue of neuralgias and dyspepsias which everywhere prevail, more or less, and are increasing in frequency.

As the day closes, we all become weary, and the body yearns for the repose and rest which only the quiet chamber can fully give. The whole system is weak, feet, fingers, arms, everything. There is not a muscle in the body which does not participate in that tiredness. The stomach is a collection of muscles; and these are called to work at each meal—and to dispose of that meal is a work of four or five hours. The more that is eaten, the more work has to be performed. Any one can see then, the striking absurdity of giving an already weak stomach four or five hours' work to do at the close of the day—of giving rest to the body by sleep, and yet keeping the stomach hard at work until nearly daylight. Its repose then, is the repose of exhaustion, and it does not wake up for breakfast, any more than the body would, if kept out of bed long past midnight. Not being waked up, it does not call for food, and there is no appetite (no "seeking," as the word literally means) for food.

But another result follows from a hearty supper, or a very late dinner: the digestion of the food requires a large amount of nervous power, leaving the other parts of the system to the same extent deficient of their natural supply, the brain in common with the others; hence, no one can sleep soundly and refreshingly after a hearty meal.

More than this, if a large meal be taken at the close of the day, when the body is weary, tired out, the stomach not only requires an extra amount of nervous power, which must be supplied at the expense of the other parts of the system, but it requires, also, an extra supply of heat, which must be supplied in the same way—and the stomach will have it, whatever mischief may result to other parts of the body, leaving the body chilly; which, in its severest forms, is called in the South a congestive chill, where the engorgement of blood is so great as to oppress the powers of life, and a stupor pervades the whole frame, out of which it never fully wakes up again, except perhaps for a single gleam at a time of partial consciousness.

Hence, the impropriety at all times, of going out into the cold air, or taking a cold bath immediately after a hearty meal, if the person is at all weakly, or is in a tired condition; for the chilliness is only increased thereby, and a fatal result is the more likely to ensue. A thousand times better would it be for this whole land, if not an atom of food was ever allowed to pass adult lips at a later hour than five o'clock in the afternoon. Such a practice, habitually and literally adhered to, would save more lives every year than are destroyed by steam, and sea, and all wars together.

